# Predicting and Monitoring Symptoms in Patients Diagnosed With Depression Using Smartphone Data: Observational Study

**DOI:** 10.2196/56874

**Published:** 2024-12-03

**Authors:** Arsi Ikäheimonen, Nguyen Luong, Ilya Baryshnikov, Richard Darst, Roope Heikkilä, Joel Holmen, Annasofia Martikkala, Kirsi Riihimäki, Outi Saleva, Erkki Isometsä, Talayeh Aledavood

**Affiliations:** 1 Department of Computer Science Aalto University Espoo Finland; 2 Department of Psychiatry University of Helsinki Helsinki Finland; 3 Helsinki and Uusimaa Hospital District Helsinki Finland; 4 School of Science Aalto University Espoo Finland; 5 City of Helsinki Mental Health Servcies Helsinki Finland; 6 University of Turku Turku Finland; 7 Turku University Central Hospital Turku Finland; 8 Finnish Institute for Health and Welfare Helsinki Finland

**Keywords:** data analysis, digital phenotyping, digital behavioral data, depression symptoms, depression monitoring, mHealth, mobile health, smartphone, mobile phone

## Abstract

**Background:**

Clinical diagnostic assessments and the outcome monitoring of patients with depression rely predominantly on interviews by professionals and the use of self-report questionnaires. The ubiquity of smartphones and other personal consumer devices has prompted research into the potential of data collected via these devices to serve as digital behavioral markers for indicating the presence and monitoring of the outcome of depression.

**Objective:**

This paper explores the potential of using behavioral data collected with smartphones to detect and monitor depression symptoms in patients diagnosed with depression. Specifically, it investigates whether this data can accurately classify the presence of depression, as well as monitor the changes in depressive states over time.

**Methods:**

In a prospective cohort study, we collected smartphone behavioral data for up to 1 year. The study consists of observations from 164 participants, including healthy controls (n=31) and patients diagnosed with various depressive disorders: major depressive disorder (MDD; n=85), MDD with comorbid borderline personality disorder (n=27), and major depressive episodes with bipolar disorder (n=21). Data were labeled based on depression severity using 9-item Patient Health Questionnaire (PHQ-9) scores. We performed statistical analysis and used supervised machine learning on the data to classify the severity of depression and observe changes in the depression state over time.

**Results:**

Our correlation analysis revealed 32 behavioral markers associated with the changes in depressive state. Our analysis classified patients who are depressed with an accuracy of 82% (95% CI 80%-84%) and change in the presence of depression with an accuracy of 75% (95% CI 72%-76%). Notably, the most important smartphone features for classifying depression states were screen-off events, battery charge levels, communication patterns, app usage, and location data. Similarly, for predicting changes in depression state, the most important features were related to location, battery level, screen, and accelerometer data patterns.

**Conclusions:**

The use of smartphone digital behavioral markers to supplement clinical evaluations may aid in detecting the presence and changes in severity of symptoms of depression, particularly if combined with intermittent use of self-report of symptoms.

## Introduction

In recent years, digital tools and algorithms have become indispensable in health care, including mental health. Data-driven technologies have the potential to renew health care, providing new avenues for personalized care, remote monitoring, and improved service access. At the same time, mental health disorders, including depression, have remained a significant concern. Depressive disorders are estimated to be the second-leading cause of life-years lost to disability worldwide [1]. Alongside markedly impacting individuals’ quality of life, depressive disorders impose a substantial economic burden, including costs to health care and societies overall due to disability, reduced employment, and impaired work productivity [2].

Psychiatric evaluations are based on clinical interviews, relying on patients’ self-reflections and recollections, which are susceptible to memory biases and subjective inaccuracies [3]. Further, the absence of definitive physiological biomarkers for mental disorders complicates accurate diagnoses and treatment [4]. Given these challenges, a growing interest has been in data-driven clinical monitoring and decision-making, supplementing subjective evaluations with objective, longitudinal, physiological, and behavioral data collected via digital devices [5]. This approach, known as digital phenotyping, involves creating a digital representation of a patient’s clinical phenotype using behavioral, social, and physiological markers. The premise of the data-driven approach lies in the inherent value of continuous monitoring, uncovering valuable insights unattainable through intermittent assessments [4].

Recent data-driven studies using devices like smartphones and activity trackers have effectively used digital behavioral data to monitor and detect participants’ depressive moods [6-8]. These studies gather sensor data to identify behavioral patterns associated with depressive disorders, such as changes in physical activity, phone usage, and sleep routines. The primary goals include differentiating between patients with depression and healthy controls, classifying mood state transitions, and predicting future mood states. Alongside passively collected data, these studies often use established self-report questionnaires as the reference standard for subjects' severity of depressive symptoms.

However, some of the studies have used limited data collection, sample sizes of fewer than 50 participants [9-11], a sample of college students [12-15], and data collected over only a few weeks [9,12,16]. Due to these limitations, it may be challenging to generalize results to either a broader population or a free-living setting. Regarding methodologies, earlier research has used smartphone sensors and data categorized as smartphone usage [9,10,14,15], GPS location data-based features [9-15,17], physical activity data or step counts [11-17], communication patterns [12,14,17], Bluetooth data [13,14], sleep data [13,15], metrics for behavior regularity [15], and physiological measurements [17]. Furthermore, studies have used several metrics for depression as the ground truth, including the 9-item Patient Health Questionnaire (PHQ-9) [9-11,17,18], a compact version of the 4-item Patient Health Questionnaire (PHQ-4) [15,19], the Montgomery and Åsberg Depression Rating Scale [16,20], and the Beck Depression Inventory-II [13,14,21]. The analysis methods used in these studies vary, encompassing correlation analysis [9,10,12], machine learning [11,13,14,16,17], and deep learning [15,16].

This paper builds on previous research, exploring the potential of using behavioral data collected with smartphones to detect and monitor depression symptoms in outpatients diagnosed with depression. Our study aims to identify digital behavioral markers indicative of depressive states and assess the accuracy of this data in detecting depression. Key markers extracted from smartphone sensors, such as the accelerometer, app usage, battery status, communication log, screen activations, and GPS location, comprise metrics like screen-on activation count, total distance traveled, average battery level, phone call count, app usage duration, and maximum acceleration. We analyzed a comprehensive dataset, gathered through smartphones, from patients with depression who have a diagnosis of either major depressive disorder (MDD), major depressive episodes with bipolar disorder (MDE|BD), or MDD with comorbidborderline personality disorder (MDD|BPD) and healthy control*s.* The focus was on distinguishing patients self-reporting moderate or more severe depression symptoms and tracking changes in reported depression levels.

## Methods

### Dataset Description

We used the data from the Mobile Monitoring of Mood (MoMo-Mood) study, a 1-year multimodal digital phenotyping study of individuals undergoing treatment for mental disorders and healthy controls [22,23]. The MoMo-Mood study recruited 164 participants from 4 different groups: healthy controls (n=31) and patients with MDD (n=85), MDD|BPD (n=27), and MDE|BD (n=21). Voluntary patients were recruited in Finland from the mood disorder outpatient treatment facilities of the Helsinki University Hospital Mood Disorder Division, Turku University Central Hospital Department of Psychiatry, and City of Espoo Mental Health Services. The patients were diagnosed with structured interviews, namely the Mini-International Neuropsychiatric Interview [24] and the Structured Clinical Interview for DSM-IV Axis II Personality Disorders [25], as having ongoing major depressive episodes. Healthy controls were collected by contacting, via email, lists of students from the University of Helsinki and Aalto University, users of student health services from these institutions, and recruiting voluntary health care personnel from Helsinki University Hospital.

Each group had more female individuals than male individuals: (1) control group, 24 female individuals and 7 male individuals; (2) MDD group, 46 female individuals and 31 male individuals; (3) MDE|BD group, 18 female individuals and 3 male individuals; and (4) MDD|BPD group, 23 female individuals and 1 male individual. On average, control group participants were older than patient group participants, with average ages as follows: (1) control group, 41.8 (SD 13.9) years, (2) MDD group, 39.0 (SD 14.2) years, (3) MDE|BD group, 37.1 (SD 10.3) years, and (4) MDD|BPD group, 28.3 (SD 6.0) years. A more detailed description is provided elsewhere [22,23].

Study participants were recruited on a rolling basis, allowing them to join and leave the research at various intervals. They were requested to stay involved in the study for 1 year. Data collection was carried out in 2 phases. In the initial 2 weeks, called the *active phase*, participants collected data continuously via personal devices (smartphones running Android operating system), bed sensors, and actigraphs, and they answered daily mood-related questions. The active phase was followed by the *passive phase,* lasting up to 1 year. During the passive phase, data collection via smartphones continued, and participants’ depression was monitored by biweekly PHQ-9 surveys prompted via the smartphone. The PHQ-9 comprises 9 questions, each scored from 0 to 3, based on the frequency of depressive symptoms over the past 2 weeks. Thus, the total score ranges from 0 to 27, with high values representing more severe depression. The passive data originate from various smartphone sensors, including accelerometers, app usage, communication, battery level and screen status logs, and GPS location data. The data were collected through the Niima data collection platform [26]. This work exclusively focuses on the passive phase of the study, which uses smartphone data and PHQ-9 survey responses. This phase was selected due to its unobtrusive data collection methods and, thus, the minimal requirement for behavioral adjustment from the participants.

### Data Preprocessing

**Figure 1:**
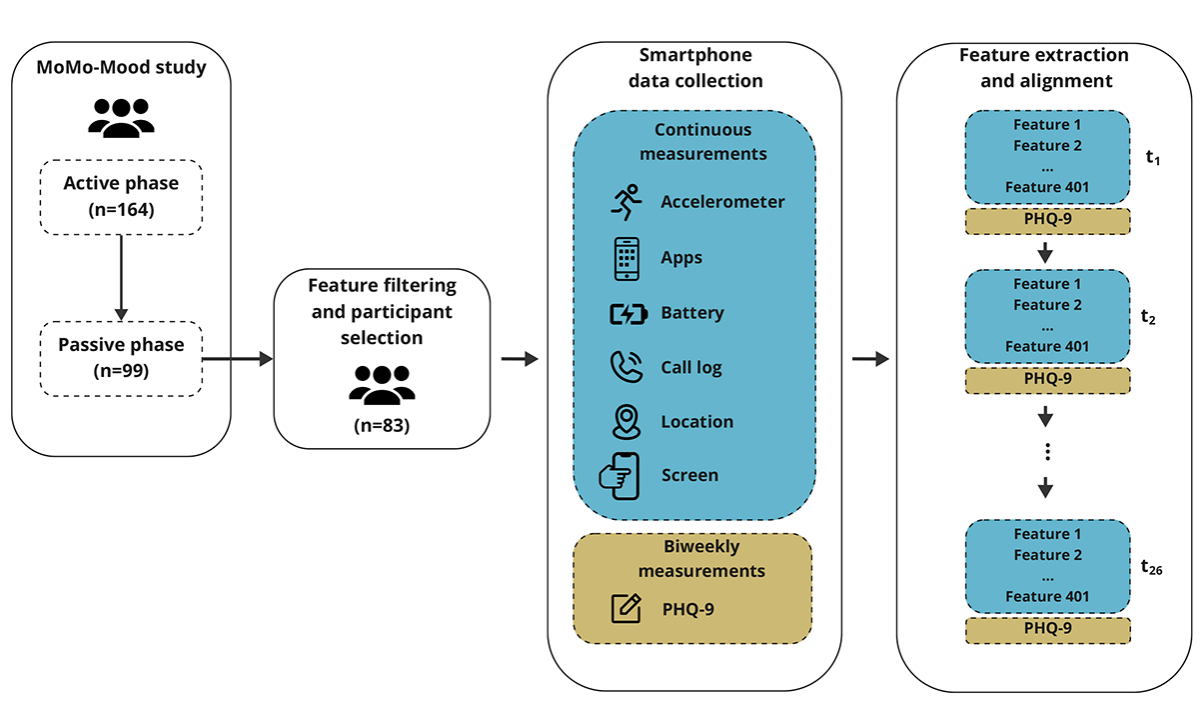
The MoMo-Mood study data collection and preparation schema. MoMo-Mood: Mobile Monitoring of Mood; PHQ-9: 9-item Patient Health Questionnaire.

**Figure 2:**
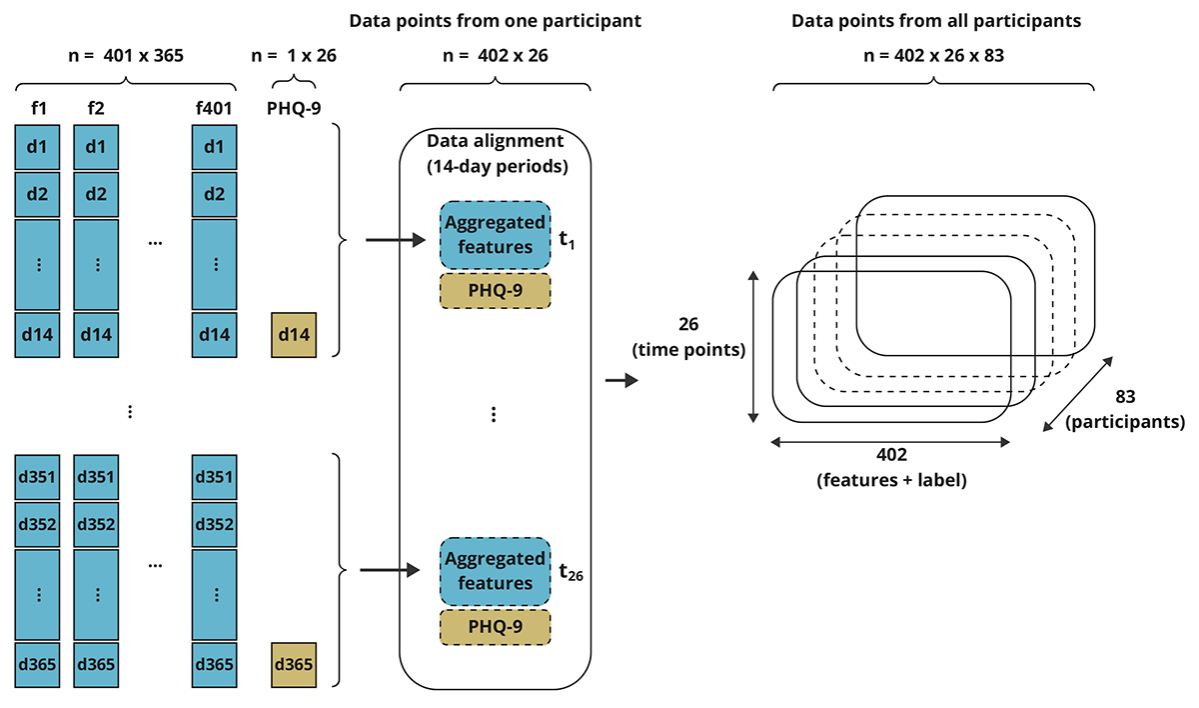
Schematics of data aggregation and alignment. PHQ-9: 9-item Patient Health Questionnaire.

We used Python and the *Niimpy* behavioral data analysis toolbox [27] for data preprocessing. We extracted 93 behavioral features from the raw data. Multimedia Appendix 1 provides a detailed description of data sources and extracted features. Furthermore, we segmented the data from the accelerometer, app usage, battery status, communication log, and smartphone screen activations into 6-hour bins (12:00 AM to 06:00 AM, 6:00 AM to 12:00 PM, 12:00 PM to 6:00 PM, and 6:00 PM to 12:00 AM). We extracted 308 additional features, resulting in a total of 401 features. The data from different sensors were resampled and averaged over 14-day periods. The data were merged with the PHQ-9 responses to align data from the preceding biweekly period with the questionnaire responses. Of the 164 participants in the active phase, 99 proceeded to the passive phase. For the analysis, we selected participants who had provided passive data for at least 14 days and had answered a PHQ-9 survey at the end of this period, yielding 83 participants. Each participant provided data for up to 1 year, yielding 26 biweekly data points. Due to missing observations and participant withdrawals from the study, 818 observations (37.9% of the possible 2158 observations) were available for further analysis. Figure 1 details the data collection and preparation for the analysis, while Figure 2 provides additional information on data aggregation and alignment.

### Statistical Analysis

#### Distributional Testing

To examine whether passively collected smartphone sensor data show differences between patient groups and control participants, we used distributional testing using the nonparametric 2-sample Kolmogorov-Smirnov test [28]. The test was chosen due to its capability to detect variations across the entire distribution, including the tails. For the test, we averaged the biweekly sampled data by participant, normalized the data, and omitted the missing values*.* For robustness against the risk of type I errors (false-positive) due to multiple comparisons, we implemented false discovery rate (FDR) correction using the Benjamini-Hochberg procedure [29] at a significance level of α=.05.

#### Correlation Analysis

We conducted a correlation analysis to assess the association between passive data features and PHQ-9 scores. We pooled passive data from all participants, omitted missing values, and applied the Spearman rank correlation coefficient to assess the strength of the relationship. Furthermore, we used FDR correction using the Benjamini-Hochberg procedure at a significance level of α=.05 to account for the multiple testing involved, controlling the expected proportion of false discoveries.

### Predictive Modeling

To achieve the research goal, we deployed supervised machine learning models for predicting both the presence of depression and state transitions of depressive states. We used a cutoff PHQ-9 depression score of 10 for binary classification analyses. Scores of 10 or higher were considered *depressed,* and scores below 10 as *nondepressed.* We chose a cutoff value of 10 because it signifies clinical depression, typically warranting a treatment plan that may include counseling, follow-up sessions, and possibly pharmacotherapy for the individual. For defining the depression state transition, we used the same threshold of 10 and the previous depression state. Each transition is paired with a specific label, used as the target variable for the depression state transition modeling. The transition definitions are presented in Table 1.

**Table 1:** Overview of depression state transition definitions and corresponding labels.

Transition description	Label
Depressed→Depressed	Remains depressed
Depressed→Nondepressed	Improves
Nondepressed→Nondepressed	Remains nondepressed
Nondepressed→Depressed	Declines

We built a machine learning pipeline using Python (version 3.10.8) and the following libraries: *scikit-learn* (version 1.2.0) [30], *extreme gradient boosting* (*XGBoost*; version 1.7.3) [31], *OPTUNA* (version 3.1.0) [32], *imbalance-learn* (version 0.11.0) [33], and *Shapley additive explanations* (*SHAP*; version 0.41.0) [34]. Initially, we partitioned our dataset into a 75%:25% train:test split, preventing data leakage by keeping each participant’s data exclusively in either the training or test set. We conducted feature prefiltering by removing features with no or low variance, many missing observations, and a high correlation with other features. We compared filtering and wrapper-based methods and embedded feature selection methods with XGBoost classifiers for feature selection. We used data missingness, variance, and cross-correlation thresholding-based feature selection for the filtering-based method and the sequential forward selection method for the wrapper-based method. Standard preprocessing was applied to selected features, comprising imputation using median values, scaling transformations, and data normalization. To address the class imbalance and improve the robustness of our classification models against overfitting to the majority class, we used the synthetic minority oversampling technique (SMOTE) [35], a method for generating synthetic minority class samples to balance the dataset. We applied SMOTE at 2 stages of the model’s development. First, the training data folds were balanced using SMOTE during the cross-validation process. We then applied SMOTE to the entire training dataset in preparation for the final model fitting. For pipeline details, refer to Multimedia Appendix 2.

**Figure 3:**
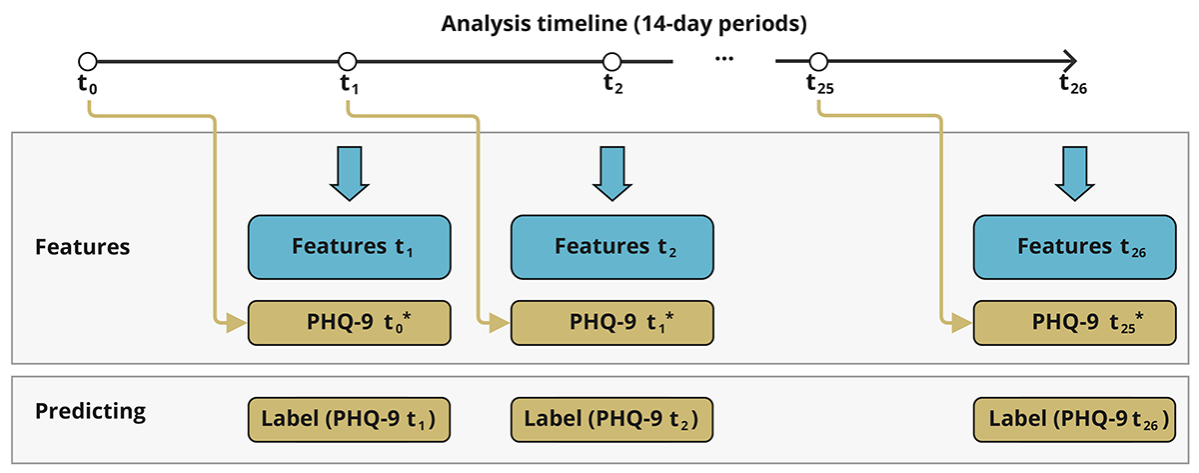
Schema for depression presence and transition prediction using passive behavioral data. An asterisk (*) depicts a model using the PHQ-9 measurement from the preceding biweekly period as a predictor. Time point t0 on the analysis timeline represents the active phase, and points from t1 to t26 represent the passive phase. PHQ-9: 9-item Patient Health Questionnaire.

In our study, we focused on the prediction task of identifying (1) the presence and (2) the state transitions of depression symptoms using passively sensed smartphone data and supervised machine learning models. Figure 3 outlines how data was used for prediction. Specifically, 3 models were examined, namely k-nearest neighbors, support vector classifier, and XGBoost, all of which are commonly used models in digital phenotyping studies [36,37]. To fine-tune feature filtering, transformation functions, classification model, and SMOTE hyperparameters, we used stratified grouped 5-fold cross-validation, using the OPTUNA framework [32]. The primary objective in the hyperparameter optimization process was to maximize the *F*_1_-score, which balances precision and recall, thereby ensuring a more reliable evaluation of model performance. We used a pruning early stopping technique, which ceases training if there is no improvement in the *F*_1_-score (our chosen validation metric). Finally, we used the test data and bootstrapping validation (using 10,000 bootstrap samples from training data) to evaluate the model performance, assessing the performances with accuracy, precision, recall, negative predictive value (NPV), and *F*_1_-scores, as defined in Multimedia Appendix 3. *F*_1_-score is a valuable metric because maximizing it ensures that both false positives (identifying a participant who is nondepressed as depressed) and false negatives (failing to identify a participant who is depressed) are minimized. High recall reflects low false-negative classification, so we emphasized its importance in model performance evaluation.

### Measuring Feature Importance

For the final part, focusing on model interpretation, we assessed the importance of features (behavioral markers) for the best-performing XGBoost models to gain insight into the underlying classification mechanisms of the model. We evaluated the importance of each feature for depression presence and the state transition classifications using SHAP values [34]. SHAP values measure each feature’s contribution to the model prediction, their relative importance compared with other features, and the significance of feature interactions.

### Ethical Considerations

The Helsinki and Uusimaa Hospital District’s Ethics Committee approved the research protocol for the MoMo-Mood study (approval number § 125/2018). A research permit was granted by Helsinki and Uusimaa Hospital District Psychiatry. This covered data streams, data collection platform security, and participants’ consent information. All data in transit were encrypted, and participant privacy was a key design value. Local IT support and the research ethics committee approved the written data security statement. Study participants were presented with full study information and data collected prior to providing written consent. The participants were allowed to withdraw whenever they chose. As remuneration for their participation, participants received 4 movie tickets at the end of the initial phase of the study.

## Results

### Descriptive Statistics

The raw data from the passive collection phase contained over 67 million data points, and 819 biweekly PHQ-9 surveys gathered data from 99 participants from 4 subgroups: 25 healthy controls, 46 patients with MDD, 16 patients with MDD|BPD, and 12 patients with MDE|BD. Participant selection, filtering, and preprocessing reduced the raw data to 327,200 data points (818 observations with 401 data features) and PHQ-9 scores to 818 observations. The resulting dataset had 83 participants, comprising 20 healthy controls, 41 patients with MDD, 12 patients with MDD|BPD, and 10 patients with MDE|BD.

### PHQ-9 Scores

**Figure 4:**
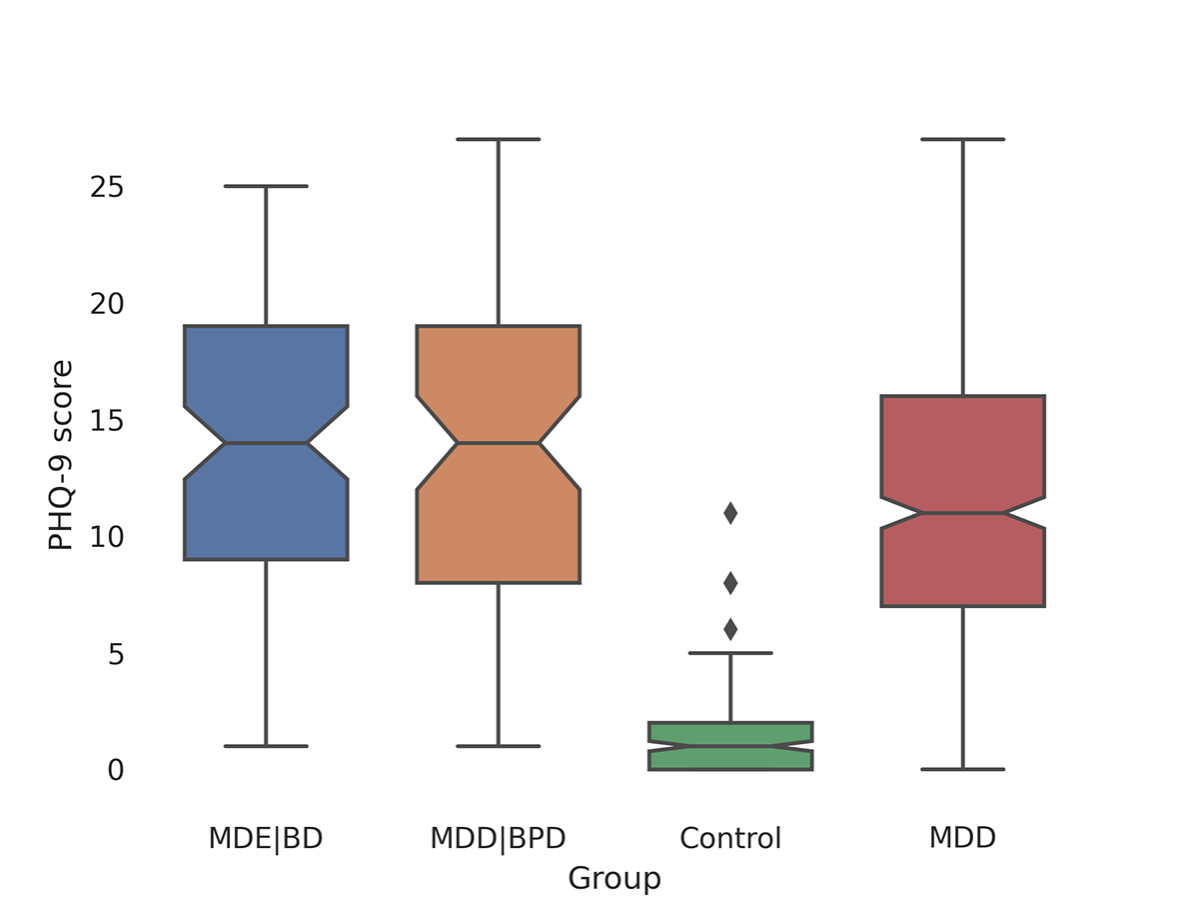
PHQ-9 score distributions for control and patient groups.To assess differences in PHQ-9 scores across various groups, we used a generalized estimating equations approach. We chose the method due to its effectiveness in dealing with correlated response data and its ability to provide robust SEs. The analysis revealed statistically significant differences in PHQ-9 scores between the control group and each of the patient groups. The significance of these differences was high, with *P*<.001 for each comparison. MDD: major depressive disorder; MDD|BPD: major depressive disorder with comorbid borderline personality disorder; MDE|BD: major depressive episodes with bipolar disorder; PHQ-9: 9-item Patient Health Questionnaire.

Most of the patients’ PHQ-9 scores during the passive data collection phase remained within the range of 5-19, representing mild to moderate clinical depression, while most control scores remained within the range of 0-4, representing no depression. The group-wise mean scores over the passive phase were as follows: control group, 1.2 (SD 1.8); MDD group, 11.9 (SD 6.7); MDE|BD group, 13.7 (SD 6.5); and MDD|BPD group, 13.8 (SD 6.6). It is noteworthy that the patient group scores predominantly represent mild to moderately severe clinical depression. Figure 4 [38] presents these differences and the distribution of PHQ-9 scores across the various groups.

**Figure 5:**
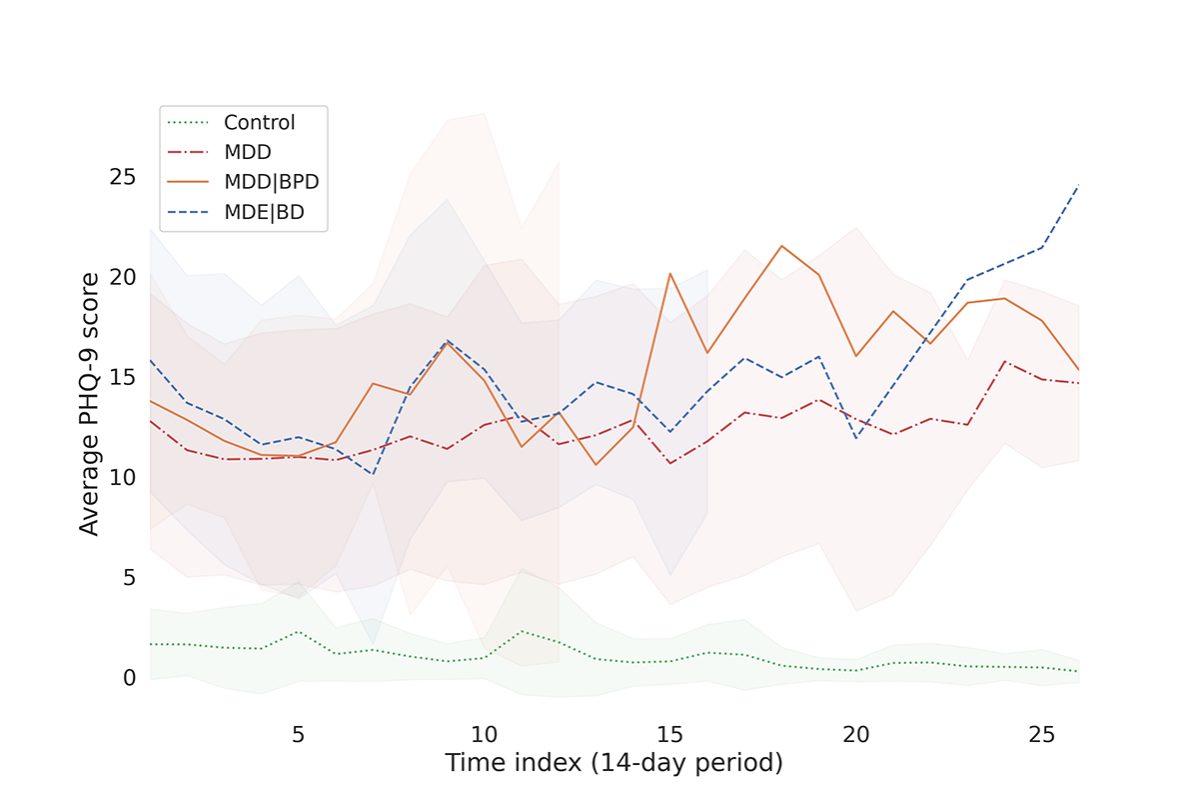
Averaged PHQ-9 score trends for controls and patient groups (standard deviations depicted by shaded regions). MDD: major depressive disorder; MDD|BPD: major depressive disorder with comorbid borderline personality disorder; MDE|BD: major depressive episodes with bipolar disorder; PHQ-9: 9-item Patient Health Questionnaire.

On average, PHQ-9 scores remain at similar levels within patient groups throughout the study, while all patient groups express a slightly decreasing trend at the beginning of the study. At the group level, MDE|BD and MDD|BPD groups exhibited more fluctuation in the scores toward the end of the study period as the number of participants within those groups decreased. Control group scores exhibited a slightly decreasing trend. Figure 5 shows the overall trends in PHQ-9 scores, averaged over each group throughout the study. It is worth noting that the number of participants decreased over time, leading to increased fluctuations in average scores.

We compared the groups by depression severity by categorizing participants using a cutoff PHQ-9 score threshold of 10. Participants with a PHQ-9 score of 10 or higher were categorized as “Depressed,” and those below 10 were categorized as “Nondepressed.” Table 2 shows the prevalence of depression severity across different patient groups.

We categorized the 818 data points into 2 groups: 347 (42.4%) participants who are depressed and 471 (57.6%) participants who are nondepressed, resulting in mildly imbalanced classes considering the classification tasks. We assessed biweekly depression state transitions for each group, as described in Table 1. Table 3 summarizes these transitions. Notably, the number of transitions for “Declines” and “Improves” was significantly lower than those for “Remains Depressed” and “Remains nondepressed.”

These results show that in the data, the state changes in depression are infrequent compared with the occurrences where the state remains the same. Here, we noticed that transition classes have a significant imbalance, as only 119 (14.5%) out of 818 state changes counted as transitions, and 699 (85.5%) were stationary. This pronounced imbalance could bias classification algorithms toward the majority class, necessitating corrective measures for reliable analysis in subsequent stages.

**Table 2:** Distribution of PHQ-9a scores by severity and group (n=818).

Group		Control	MDE|BD^b^	MDD|BPD^c^	MDD^d^	Total
Depression severity, n (%)						
	Depressed	1 (0.1)	65 (7.9)	50 (6.1)	231 (28.2)	347 (42.4)
	Nondepressed	204 (24.9)	36 (4.4)	24 (2.9)	207 (25.3)	471 (57.6)
	Total	205 (25.1)	101 (12.3)	74 (9)	438 (53.5)	818 (100)

^a^PHQ-9: 9-Item Patient Health Questionnaire.

^b^MDE|BD: major depressive episodes with bipolar disorder.

^c^MDD|BPD: major depressive disorder with comorbid borderline personality disorder.

^d^MDD: major depressive disorder.

**Table 3:** Depression state transition counts for the control group and each patient group.

Group			Control		MDE|BD^a^		MDD|BPD^b^		MDD^c^		Total
Transition, n											
	Declines	1		9		5		34		49	
	Improves	1		15		11		43		70	
	Remains depressed	0		57		42		222		320	
	Remains nondepressed	203		20		17		139		379	
	Total	205		101		74		438		818	

^a^MDE|BD: major depressive episodes with bipolar disorder.

^b^MDD|BPD: major depressive disorder with comorbid borderline personality disorder.

^c^MDD: major depressive disorder.

### Data Completeness

Participant compliance and, thus, data completeness decreased as the study’s passive phase progressed. PHQ-9 survey answer compliance dropped below 70% after 6 weeks (3 biweekly periods) had passed, and after that, it continued to decline steadily. For further details, refer to Multimedia Appendix 4. Passive data collection compliance shows a pattern similar to answering the PHQ-9 survey. Most of the missing data occurred due to the participant dropping out of the study, while some participants had gaps in data collection. Notably, only a few participants remained in the study for the entire year. Also, the data collection for participants was incomplete due to missing features.

### Statistical Analysis

Two-sample distributional testing using a 2-sample Kolmogorov-Smirnov test identified 20 significant features (5%), with *P* values ranging from .0045 to .0497. However, after applying the FDR correction for multiple comparisons at a significance level ⍺=.05, none of these features were statistically significant; thus, we found no evidence for patient group behavioral data differing from control data. For further details, see Table S1 in Multimedia Appendix 5.

Correlation analysis between the behavioral features and PHQ-9 scores using Spearman ranked correlation and FDR correction for multiple comparisons at significance level ⍺=.05 resulted in 32 (8%) out of 401 features exhibiting statistically significant correlations. The majority (18/32, 56%) of the correlations were very weak (absolute value from 0 to 0.19), and the rest (14/32, 44%) were weak (absolute value from 0.2 to 0.39). For more information, refer to Table S2 in Multimedia Appendix 5.

### Depression Presence Classification

We used 2 distinct approaches for classifying the presence of depression. The initial approach treated all biweekly aggregated passive data features (aligned with corresponding biweekly PHQ-9 scores) as independent observations. Using the XGBoost classifier with filter-based feature selection, we achieved the highest accuracy of 66% (95% CI 56%-70%) and an *F*_1_-score of 0.66 (95% CI 0.5-0.7). The performance comparison of various classifiers and feature selection methods is detailed in Table S1 in Multimedia Appendix 6, while Table S2 in Multimedia Appendix 6 provides a comprehensive summary of the model’s performance.

For the second modeling approach, we included the measured PHQ-9 score from the previous biweekly period as a predictor in the model. Model performance improves notably after adding the predictor. XGBoost classifier with a filtering-based feature selection method achieved the best accuracy of 82% (95% CI 80%-84%) and a corresponding *F*_1_-score of 0.82 (95% CI 0.80-0.85) across the test data of 208 samples.

This classifier outperformed the other classifiers (k-nearest neighbor and support vector classifier) by a small margin. The “Nondepressed” class (99 samples) achieved a precision of 0.80 and recall of 0.83, with an NPV of 0.84 and an *F*_1_-score of 0.81, reflecting balanced performance. The “Depressed” class (109 samples) had a slightly higher precision of 0.84, a recall of 0.81, an NPV of 0.80, and an *F*_1_-score of 0.82, indicating a similar level of predictive accuracy to the “Nondepressed” class. Both macro- and weighted averages across precision, recall, *F*_1_-score, and NPV are 0.82, demonstrating consistent performance in detecting both the presence and absence of depression. Table S3 in Multimedia Appendix 6 summarizes the performance of selected classifiers and feature selection methods. Table 4 summarizes the XGBoost classifier's performance, and Figure S1 in Multimedia Appendix 6 presents the receiver operating characteristic curve for the classifier.

**Table 4:** XGBoost^a^ model performance metrics for depression presence classification (using previous PHQ-9^b^ score as a predictor).

Metric			Precision		Recall		NPV^c^		*F*_1_-score		Support, n
Class											
	Nondepressed	0.80		0.83		0.84		0.81		99	
	Depressed	0.84		0.81		0.80		0.82		109	
Averages											
	Macroaverage	0.82		0.82		0.82		0.82		208	
	Weighted average	0.82		0.82		0.82		0.82		208	

^a^XGBoost: extreme gradient boosting.

^b^PHQ-9: 9-item Patient Health Questionnaire.

^c^NPV: negative predictive value.

### Depression State Transition Classification

For depression state transition classification, we used the XGBoost classifier with feature filtering since it performed best in the depression presence classification. The model was able to classify relatively well the cases where a participant’s state remains the same, while the accuracy is considerably lower for cases where the state changes. Applying SMOTE’s synthetic oversampling technique to alleviate class imbalance significantly increased the recall of the minority classes (depression transitions for “Declines” and “Increases”). The model achieved an accuracy of 75% (95% CI 72%-76%) and a corresponding *F*_1_-score of 0.67 (95% CI 0.63-0.69). Table 5 summarizes the model validation results for each transition type. For the transition of “Declines,” the model shows high NPV (0.98) but lower precision (0.34). It indicates that while the model reliably identifies cases where the state will not decline, it is less accurate at correctly identifying the cases where it declines. The recall is 0.76, leading to an *F*_1_-score of 0.47, signifying unbalanced classification performance. “Increases” shows a similar pattern with high NPV (0.96) and moderate recall (0.74) but lower precision (0.46), resulting in an *F*_1_-score of 0.57, also indicating unbalanced classification performance. For the “Remains Depressed” and “Remains nondepressed” states, the model exhibits higher precision (0.93 and 0.95, respectively) and NPV (0.86 and 0.83, respectively), along with recall rates of 0.72 and 0.77, leading to a more balanced performance with *F*_1_-scores of 0.81 and 0.85. The macroaverage *F*_1_-score of 0.67, compared with the overall accuracy of 0.75, reflects the effect of class imbalance on the model's performance. Further, Figures S2 and S3 in Multimedia Appendix 6 display classification outcomes for the test data and a multiclass receiver operating characteristic curve for the XGBoost classifier.

The results show the model’s ability to classify most cases correctly. With an overall accuracy of 75%, the model effectively balances precision across different cases. These findings demonstrate the model’s potential for predicting depression state transitions, leveraging smartphone-sensed behavioral data and self-reported PHQ-9 scores.

**Table 5:** XGBoost^a^ model performance metrics for depression state transition classification (using previous PHQ-9^b^ score as a predictor).

Metric			Precision		Recall		NPV^c^		*F*_1_-score		Support, n
Transition											
	Declines	0.34		0.76		0.98		0.47		17	
	Increases	0.46		0.74		0.96		0.57		23	
	Remains depressed	0.93		0.72		0.86		0.81		74	
	Remains nondepressed	0.95		0.77		0.83		0.85		94	
Averages											
	Macroaverage	0.67		0.75		0.91		0.67		208	
	Weighted average	0.84		0.75		0.87		0.77		208	

^a^XGBoost: extreme gradient boosting.

^b^PHQ-9: 9-Item Patient Health Questionnaire.

^c^NPV: negative predictive value.

### Feature Importance Analysis Using SHAP Values

In our analysis of feature importance for classification of the presence of depression and depression state transition, we evaluated the relative significance of different features by examining the SHAP values in the best-performing XGBoost models. In summary, our findings highlight the previous PHQ-9 score as the most impactful feature when included in the model. For depression presence classification, additional significant features include smartphone screen status, app usage, battery level, phone call, and location–related information. In addition to the previous PHQ-9 score for state transition classification, screen status, location–, battery level– and accelerometer–related features stand out as the most important. Conversely, app usage and communication–related features had a limited impact on the models.

The importance of the previous PHQ-9 score implies that the depression scores are autocorrelated, thus reflecting future depression levels. Smartphone screen status (e.g., screen on and off event counts) reveals users' interaction with the device, showing usage frequency and patterns. Similarly, battery level indicates phone usage, reflecting battery drains and charges. App usage features (especially apps labeled as leisure, sports, and social media) suggest behavioral patterns related to such activities as watching movies or listening to music, exercising, and communicating via social media. Finally, accelerometer-related features reveal physical activity and mobility patterns.

Figures S1-S3 in Multimedia Appendix 7 present the most important features of these classifications. Specifically, Figure S1 in Multimedia Appendix 7 illustrates the important features of depression presence classification without considering the previous biweekly PHQ-9 scores. Conversely, Figure S2 in Multimedia Appendix 7 shows the results for the model, including these scores as a predictor. Finally, Figure S3 in Multimedia Appendix 7 explores features pertinent to depression state transition classification.

## Discussion

### Principal Findings

Our analysis encompassed passively sensed digital behavioral data, which we compared against actively collected PHQ-9 survey data. Using the generalized estimating equation approach, we discovered a statistically significant difference in PHQ-9 score distributions between the control and patient groups. It is important to note that some patients likely experienced recovery after recruitment for the study, potentially lessening the severity of symptoms reflected in their PHQ-9 scores. Consequently, our data could underrepresent the depression severity spectrum, particularly among patients with more severe depression.

After adjusting for multiple comparisons, distributional testing on behavioral features revealed no significant differences between control and patient groups. This finding suggests that the differences in behavioral data at the group level are minimal. Therefore, our study implies that detecting these subtle differences might require larger sample sizes or alternative statistical methodologies that can leverage hierarchical structures and temporal correlations.

Correlation analysis identified 32 behavioral features with weak or very weak correlations with PHQ-9 scores, predominantly involving smartphone screen interaction (18 features) and accelerometer data (14 features). Despite most features showing no significant correlation with PHQ-9 scores, their potential value in classification tasks remains, especially considering possible nonlinear relationships or interactions with other features.

For the depression prediction tasks, we found that the XGBoost classifier with filtering-based feature selection performed the best in discriminating between participants who are depressed and nondepressed, achieving 66% accuracy. The accuracy increased to 82% when we added the PHQ-9 score from the previous biweekly period as a predictor. The difference implies the importance of the temporal structure of the data. Therefore, we propose to include temporal information in future analyses to improve the accuracy. Further, for clinical monitoring applications, information about participants’ depression histories should be available, providing the temporal context necessary to enhance the model's predictive accuracy.

Furthermore, our results show that the XGBoost classifier, combined with filter-based feature selection and PHQ-9 measurement from the previous biweekly monitoring period as a predictor, can differentiate mood state transitions with a classification accuracy of 75%. While promising, this accuracy level suggests room for further improvement in the model’s performance. Like the depression presence classification, we suggest using more comprehensive methods, personalized models, and temporal information. Additionally, we suspect that the data’s limited sample size and sparsity of transition events hinder the classification performance. Therefore, model development should benefit from a larger sample.

Finally, feature importance analysis revealed insights into the key features of depression prediction models. The most significant predictor for detecting and classifying depression presence was previous biweekly PHQ-9 scores, complemented by features related to accelerometer, app usage, battery level, location, and screen events. The results emphasize the significance of daily behavioral patterns and time-of-day distinctions (morning, afternoon, evening, and nighttime) in accurately predicting depression. Interestingly, some features were identified by both the correlation and feature importance analyses for classifier models. While the methods and objectives of these analyses differ, the consistency in identifying the same key features across both approaches implies their potential relevance in depression prediction.

### Comparison With Previous Studies

Our study aligns methodologically with previous research using validated depression assessments and analyzing passively collected smartphone behavioral features. Also, it focuses on statistical inference and machine learning techniques to classify depression among participants and distinguish participants based on behavioral data. Additionally, the identified important features are consistent with earlier research reporting features related to phone usage [9,10,14,15], physical activity [11-17], and location data [9-15,17]. By contrast, the importance of features related to communication [12,14,17] were slightly underrepresented in our analysis.

Our classification results are numerically comparable to previous studies using machine learning methods with smartphone data for depression detection. Using a cohort of college students, Chikersal et al [14] achieved an 85% accuracy and an *F*_1_-score of 0.82 in the postsemester depression detection task. They also achieved an 85% accuracy and an *F*_1_-score of 0.80 in detecting a change in the depression state task. Similarly, Wang et al [15] used machine learning and deep learning models to detect depression using a subset of smartphones, also from a cohort of college students, achieving an *F*_1_-score of 0.65 using a machine learning model and an *F*_1_-score of 0.7 using deep learning.

However, our study differentiates itself by including a diverse cohort of real outpatients, clinically diagnosed with structured interviews, alongside control participants, thereby offering a broader perspective on depression. Additionally, the data are collected over an extended period in a naturalistic setting, enhancing the reliability of the findings. Unlike other studies that often focus on student populations, it demonstrates the feasibility of digital behavioral monitoring in real outpatients. Furthermore, it excludes certain data features like physiological measurements and social engagement metrics. Lastly, the study does not aim to predict future depressive states, setting it apart from other predictive modeling efforts in the field.

### Limitations

While this research yields insightful outcomes, it is crucial to acknowledge certain limitations. First, dropouts and missing data increased substantially after the first 3 biweekly periods. Some participants provided data only for 1 biweekly period, limiting our model’s ability to capture patient symptom fluctuations. Second, our analysis does not fully account for the hierarchical and autocorrelational structure of the data. We rely on simplified analysis, using aggregated features and pooled participants, resulting in the loss of available information. Finally, our study does not accommodate external factors that might impact the participants’ behavior patterns and mood states. Given that the data collection partially took place during the COVID-19 era, factors such as social isolation could have played a role in changing the behavior patterns and emotional states of participants.

### Recommendations for Future Work

This study lays the groundwork for multiple future research endeavors. A direct expansion of our work would be the implementation of personalized models designed to predict the depression state of individuals. These personalized models, which incorporate both group and participant variations and sample-level information, have demonstrated improved accuracy in depression classification tasks [39]. Furthermore, we recommend fully using the temporal structure of the data in classification tasks. Given the inherent variability in symptomatic periods among patients with depression, analyzing temporal patterns and trends from longitudinal data could offer a more accurate representation of their evolving mental states than single-point estimates. We also encourage the exploration of deep learning models in future studies, as these models tend to surpass conventional machine learning methods in predictive accuracy [15,16]. However, due to their complexity and less clear interpretability relative to more traditional methods, we suggest not starting with these models at the outset, instead gradually incorporating them into the analysis. Lastly, to address the challenges posed by the unbalanced dataset in our study, we suggest collecting additional data to enhance the robustness and generalizability of future research findings.

### Conclusions

In summary, this study demonstrates the potential of using smartphone-sensed behavioral data for monitoring depression symptoms, thereby paving the way for personalized and more effective mental health care. The results contribute to an expanding body of evidence supporting the integration of data-driven methods into mental health services. These insights may complement and enhance clinical practices, supplementing conventional diagnostic and monitoring approaches.

## Data Availability

Due to the fact that the data are highly sensitive, the collected data cannot be shared with researchers outside of our consortium. Our research permit does not allow the free availability of these data.

## Appendix

Multimedia Appendix 1 Summary of data sources and extracted features for each sensor.

Multimedia Appendix 2 The machine learning pipeline for depression presence and state transition classification.

Multimedia Appendix 3 Definitions for key performance metrics accuracy, precision, recall, negative predictive value, and F1-score.

Multimedia Appendix 4 The percentage of participants providing PHQ-9 data over time by group. PHQ-9: 9-item Patient Health Questionnaire.

Multimedia Appendix 5 Kolmogorov-Smirnov test results for behavioral data distribution differences and Spearman rank correlation test results between behavioral data features and PHQ-9 scores. PHQ-9: 9-item Patient Health Questionnaire.

Multimedia Appendix 6 Performance comparison of depression presence classification models. Performance metrics for depression presence classification: nondepressed versus depressed. Comparative performance of depression presence classification models with biweekly PHQ-9 score as a predictor. ROC curves for depression presence classification and different depression transition classifications. Confusion matrix for depression state transition classification. PHQ-9: 9-item Patient Health Questionnaire; ROC: receiver operating characteristic.

Multimedia Appendix 7 Key features in depression presence XGBoost classification model based on SHAP values. Key features in depression presence classification XGBoost model with preceding biweekly PHQ-9 score as a predictor. Key features in depression state transition classification XGBoost model with previous PHQ-9 score. PHQ-9: 9-item Patient Health Questionnaire; SHAP: Shapley additive explanations; XGBoost: extreme gradient boosting.

## Abbreviations

FDR: false discovery rate
MDD: major depressive disorder
MDD|BPD: major depressive disorder with comorbid borderline personality disorder
MDE|BD: major depressive episodes with bipolar disorder
MoMo-Mood: Mobile Monitoring of Mood
NPV: negative predictive value
PHQ-4: 4-item Patient Health Questionnaire
PHQ-9: 9-item Patient Health Questionnaire
SMOTE: synthetic minority over-sampling technique
SHAP: Shapley additive explanations
XGBoost: extreme gradient boosting
