# Supplementary material for: Predicting and Monitoring Symptoms in Patients Diagnosed With Depression Using Smartphone Data: Observational Study
Source: J Med Internet Res. 2024 Dec 3;26:e56874. doi: 10.2196/56874 (PMC11653032; doi:10.2196/56874)
Supplement: Multimedia Appendix 1 [file jmir_v26i1e56874_app1.docx]

## **Multimedia Appendix 1**

**Table S1.** Summary of data sources and extracted features for each sensor.

| Sensor | Extracted features |
| --- | --- |
| Accelerometer | Acceleration magnitude, magnitude max, min, mean and standard deviation, measurement count |
| Application | Application class, count, duration |
| Battery | Battery level mean, median and standard deviation, battery shutdown time, battery discharge |
| Communication | Total call duration, call duration mean, median and standard deviation, call count, outgoing-incoming call ratio, SMS count |
| Location | *Distance-based features:* total distance, variance, log variance, average speed, speed variance, max speed, location bin count.  *Significant place-related features:* static point count, moving point count, static bin count, max distance from home, number of significant places, number of rarely visited places, number of transitions between significant places, bin count in top1, top2, top3, top4, and top5 cluster, normalized entropy |
| Screen | Screen off timestamp, screen event count, screen event duration, screen event duration max, mean, min, and standard deviation, screen first unlock timestamp |
| Survey | PHQ-9 score |

Table S1 summarizes the data sources, specifically the sensors used, and the corresponding features extracted from each sensor. For a detailed reference, see Niimpy documentation <https://github.com/digitraceslab/niimpy> (“Niimpy: Behavioral data analysis”, 2023).
