# Supplementary material for: Predicting and Monitoring Symptoms in Patients Diagnosed With Depression Using Smartphone Data: Observational Study
Source: J Med Internet Res. 2024 Dec 3;26:e56874. doi: 10.2196/56874 (PMC11653032; doi:10.2196/56874)
Supplement: Multimedia Appendix 2 [file jmir_v26i1e56874_app2.docx]

## **Multimedia Appendix 2**

**Figure S1.** The machine learning pipeline for depression presence and state transition classification.


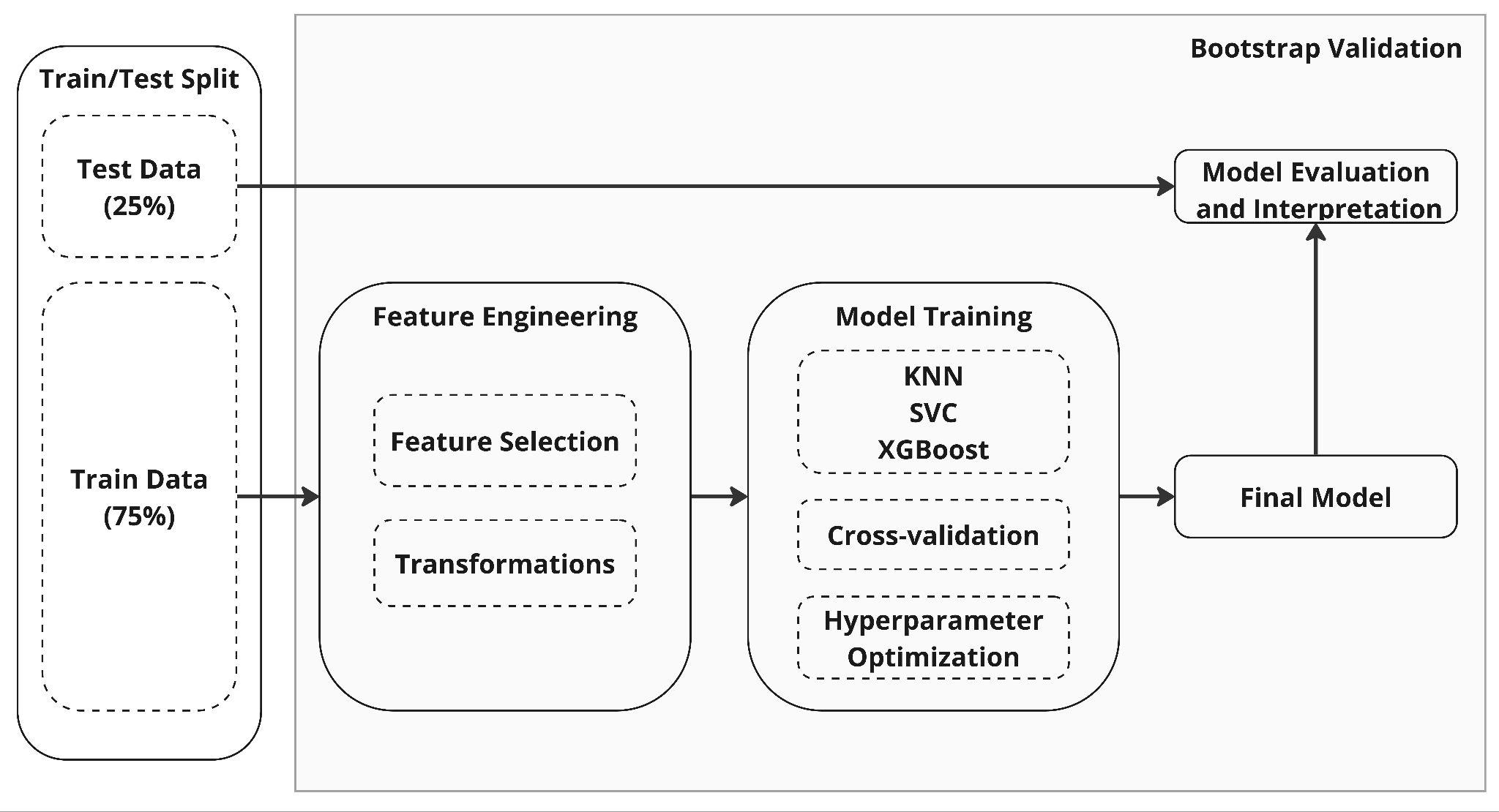


Figure S1 depicts the machine learning pipeline starting with a 75%/25% train/test split. Feature engineering includes feature filtering (removing features with low variance, high correlation with other features, and high amount of missing values), feature selection (filtering and wrapper-based, and embedded methods), and transformations (encoding, imputation, and scaling). Models (KNN, SVC, and XGBoost) were trained using stratified grouped 5-fold cross-validation with hyperparameter optimization within the Optuna framework, optimizing the F1-score. The final model was evaluated using test data and bootstrapping (10,000 bootstrapping samples) validation to obtain confidence intervals for performance metrics (accuracy, precision, recall, NPV, and F1-score).
