# Supplementary material for: Predicting and Monitoring Symptoms in Patients Diagnosed With Depression Using Smartphone Data: Observational Study
Source: J Med Internet Res. 2024 Dec 3;26:e56874. doi: 10.2196/56874 (PMC11653032; doi:10.2196/56874)
Supplement: Multimedia Appendix 3 [file jmir_v26i1e56874_app3.docx]

## **Multimedia Appendix 3**

**Table S1.** Definitions for key performance metrics accuracy, precision, recall, negative predictive value, and F1-score.

| ***Accuracy*** is the proportion of correct predictions out of all the predictions made and is defined as  $Accuracy=\frac{true positives \left( TP \right) + true negatives \left( TN \right)}{all predictions},$  where TP indicates the number of correctly identified positive instances and TN is the number of correctly identified negative instances.  ***Precision*** (also known as ***positive predictive value*** ***(PPV))*** measures the accuracy of the positive predictions. It is defined as  $Precision=\frac{true positives \left( TP \right)}{false positives \left( FP \right) + true positives \left( TP \right)},$    where FP is the number of incorrectly identified negative instances.  ***Recall*** (also known as ***sensitivity***) measures the model's ability to detect true positives and is defined as  $Recall=\frac{true positives \left( TP \right)}{true positives \left( TP \right) + false negatives \left( FN \right)}$,  where FN indicates the number of incorrectly identified positive instances.  ***The negative predictive value***  ***(NPV)*** measures the accuracy of negative predictions. It is defined as  $Negative Predictive Value=\frac{true negatives \left( TN \right)}{true negatives \left( TN \right) + false negatives \left( FN \right)}$ .  ***F1-score*** is defined as the harmonic mean of precision and recall scores  $F1=2\times\frac{precision \times recall}{precision + recall}$ |
| --- |

Table S1 explicates the calculation of these metrics based on the model's classifications: true positives (TP) – positive instances correctly predicted, false positives (FP) – negative instances incorrectly classified as positive, false negatives (FN) – positive instances incorrectly classified as negative, and true negatives (TN) – negative instances correctly predicted.
