# Supplementary material for: Predicting and Monitoring Symptoms in Patients Diagnosed With Depression Using Smartphone Data: Observational Study
Source: J Med Internet Res. 2024 Dec 3;26:e56874. doi: 10.2196/56874 (PMC11653032; doi:10.2196/56874)
Supplement: Multimedia Appendix 4 [file jmir_v26i1e56874_app4.docx]

## **Multimedia Appendix 4**

## **Table S1.** The percentage of subjects providing PHQ-9 data over time by group.

| group | | control (%) | BPD (%) | MDD (%) | BD (%) | total (%) |
| --- | --- | --- | --- | --- | --- | --- |
| **time (14-day period)** | |  |  |  |  |  |
|  | 1 | 100 | 100 | 100 | 100 | 100 |
|  | 2 | 56.2 | 56.2 | 80.4 | 83.3 | 75.8 |
|  | 3 | 37.5 | 37.5 | 80.4 | 75 | 69.7 |
|  | 4 | 50 | 50 | 73.9 | 83.3 | 65.7 |
|  | 5 | 31.2 | 31.2 | 69.6 | 75 | 58.6 |
|  | 6 | 31.2 | 31.2 | 67.4 | 75 | 55.6 |
|  | 7 | 37.5 | 37.5 | 58.7 | 75 | 52.5 |
|  | 8 | 25 | 25 | 52.2 | 33.3 | 43.4 |
|  | 9 | 12.5 | 12.5 | 47.8 | 41.7 | 38.4 |
|  | 10 | 18.8 | 18.8 | 45.7 | 33.3 | 36.4 |
|  | 11 | 12.5 | 12.5 | 41.3 | 33.3 | 33.3 |
|  | 12 | 18.8 | 18.8 | 39.1 | 25 | 33.3 |
|  | 13 | 12.5 | 12.5 | 39.1 | 25 | 31.3 |
|  | 14 | 12.5 | 12.5 | 41.3 | 25 | 29.3 |
|  | 15 | 12.5 | 12.5 | 28.3 | 25 | 25.3 |
|  | 16 | 6.2 | 6.2 | 28.3 | 25 | 24.2 |
|  | 17 | 12.5 | 12.5 | 26.1 | 25 | 24.2 |
|  | 18 | 12.5 | 12.5 | 28.3 | 25 | 24.2 |
|  | 19 | 6.2 | 6.2 | 17.4 | 16.7 | 17.2 |
|  | 20 | 12.5 | 12.5 | 15.2 | 8.3 | 15.2 |
|  | 21 | 6.2 | 6.2 | 13 | 8.3 | 12.1 |
|  | 22 | 6.2 | 6.2 | 15.2 | 0 | 12.1 |
|  | 23 | 6.2 | 6.2 | 15.2 | 0 | 12.1 |
|  | 25 | 6.2 | 6.2 | 6.5 | 8.3 | 9.1 |
|  | 26 | 6.2 | 6.2 | 10.9 | 0 | 10.1 |

## Table S1 shows the percentage of users within each group answering the PHQ-9 questionnaire at each time point during the study. The last column represents the total proportion of users across all groups. The percentages approximate the proportion of subjects remaining within the study. Notably, some percentages are higher than the preceding ones due to intermittent missing values.
