# Supplementary material for: Predicting and Monitoring Symptoms in Patients Diagnosed With Depression Using Smartphone Data: Observational Study
Source: J Med Internet Res. 2024 Dec 3;26:e56874. doi: 10.2196/56874 (PMC11653032; doi:10.2196/56874)
Supplement: Multimedia Appendix 5 [file jmir_v26i1e56874_app5.docx]

## **Multimedia Appendix 5**

**Table S1.** Kolmogorov-Smirnov test results for behavioral data distribution differences.

| Group | | MDE\|BD |  | MDD\|BPD |  | MDD |  |
| --- | --- | --- | --- | --- | --- | --- | --- |
|  | | P value | Corrected P value | P value | Corrected P value | P value | Corrected P value |
| **Feature** | |  |  |  |  |  |  |
|  | Morning acceleration standard deviation | .01 | .36 | .03 | .94 | .034 | >.999 |
|  | Leisure applications usage count | < .001 | .69 | .38 | >.999 | .31 | .87 |
|  | Sports applications usage count | .72 | >.999 | .004 | .37 | .14 | .87 |
|  | Afternoon leisure applications usage count | .04 | .8 | .47 | >.999 | .18 | .87 |
|  | Morning leisure applications usage count | .03 | .61 | .08 | >.999 | .06 | >.999 |
|  | Nighttime security applications usage count | .48 | >.999 | .02 | .77 | .6 | >.999 |
|  | Afternoon mean battery level | .17 | .92 | .53 | >.999 | .02 | >.999 |
|  | Morning mean battery level | .02 | .43 | .31 | >.999 | .05 | >.999 |
|  | Afternoon acceleration count | .17 | .92 | .02 | .77 | .17 | .87 |
|  | Nighttime acceleration count | .01 | .38 | .003 | .37 | .06 | >.999 |
|  | Evening maximum acceleration | .57 | >.999 | .05 | >.999 | .13 | .87 |
|  | Morning maximum accelration | .02 | .39 | .03 | .94 | .02 | >.999 |
|  | Afternoon minimum acceleration | .01 | .83 | .18 | >.999 | .59 | >.999 |
|  | Evening minimum acceleration | .01 | .36 | .21 | >.999 | .62 | >.999 |
|  | Nighttime minimum acceleration | .09 | .87 | .15 | >.999 | .01 | >.999 |
|  | Afternoon mean acceleration | .09 | .87 | .01 | .59 | .03 | >.999 |
|  | Nighttime mean acceleration | .14 | .9 | .04 | >.999 | .009 | >.999 |
|  | Morning screen use  count | .04 | .74 | .07 | >.999 | .11 | .87 |
|  | Leisure applications usage duration | .01 | .36 | .79 | >.999 | .30 | .87 |
|  | Sports applications usage duration | .72 | >.999 | .009 | .59 | .17 | .87 |

Table S1 presents the Kolmogorov-Smirnov(KS) -test results, assessing the behavioral data distribution differences between the control and patient groups, MDE|BD, MDD|BPD, and MDD. Features are included in this table if they demonstrate a statistically significant difference between at least one of the three groups and the control group. Importantly, although differences are observed (20 features out of 401), none of the features reached statistical significance (p < .05) after controlling the false discovery rate with the Benjamini-Hochberg procedure. The presented features were extracted from the smartphone accelerometer, application usage, battery level, and screen events data.

**Table S2.** Spearman rank correlation test results between behavioral data features and PHQ-9 Scores.

| Feature | Spearman correlation | P value | Corrected  P value |
| --- | --- | --- | --- |
| Nighttime screen usage count | 0.17 | <.001 | <.001 |
| Nighttime acceleration std | 0.14 | <.001 | <.001 |
| Morning minimum acceleration | 0.13 | <.001 | <.001 |
| Afternoon screen usage count | 0.11 | <.001 | <.001 |
| Screen-on total duration | 0.11 | <.001 | .01 |
| Nighttime screen-off count | 0.1 | .01 | .01 |
| Nighttime screen usage duration std | 0.09 | .01 | .03 |
| Morning screen usage count | -0.08 | .02 | .04 |
| Evening screen usage duration std | -0.11 | <.001 | .01 |
| Morning maximum screen usage duration | -0.11 | <.001 | <.001 |
| Evening minimum acceleration | -0.12 | <.001 | .01 |
| Evening acceleration count | -0.12 | <.001 | .01 |
| Morning screen-off count | -0.12 | <.001 | <.001 |
| Morning screen usage duration std | -0.13 | <.001 | <.001 |
| Afternoon maximum acceleration | -0.14 | <.001 | <.001 |
| Morning acceleration count | -0.16 | <.001 | <.001 |
| Acceleration count | -0.19 | <.001 | <.001 |
| Afternoon acceleration | -0.2 | <.001 | <.001 |
| Afternoon minimum screen-off duration | -0.22 | <.001 | <.001 |
| Morning median screen-off duration | -0.22 | <.001 | <.001 |
| Evening acceleration | -0.22 | <.001 | <.001 |
| Afternoon maximum screen-off duration | -0.22 | <.001 | <.001 |
| Nighttime acceleration count | -0.23 | <.001 | <.001 |
| Morning maximum screen-off duration | -0.23 | <.001 | <.001 |
| Afternoon median screen-off duration | -0.24 | <.001 | <.001 |
| Morning screen-off duration std | -0.27 | <.001 | <.001 |
| Morning maximum acceleration | -0.27 | <.001 | <.001 |
| Total screen-off duration | -0.29 | <.001 | <.001 |
| Morning acceleration | -0.3 | <.001 | <.001 |
| Nighttime acceleration | -0.33 | <.001 | <.001 |
| Evening maximum screen-off duration | -0.38 | <.001 | <.001 |
| Nighttime minimum acceleration | -0.38 | <.001 | <.001 |

Table S2 presents the Spearman rank correlation test results between the behavioral data features and the biweekly assessed PHQ-9 scores. Only the features exhibiting statistically significant correlation (32 out of 401) are shown in the table. Column ‘correlation’ shows the test correlation coefficient, ‘P value’ corresponding P-value, and ‘corrected P value’ the P-values after applying the Benjamini-Hochberg procedure to control type-I errors due to multiple comparisons at the significance level of α=.05. These presented features are extracted from smartphone accelerometer and screen activations. The correlation coefficients range from -0.38 to 0.17, thus ranging from low to moderate correlations.
