# Supplementary material for: Predicting and Monitoring Symptoms in Patients Diagnosed With Depression Using Smartphone Data: Observational Study
Source: J Med Internet Res. 2024 Dec 3;26:e56874. doi: 10.2196/56874 (PMC11653032; doi:10.2196/56874)
Supplement: Multimedia Appendix 6 [file jmir_v26i1e56874_app6.docx]

## **Multimedia Appendix 6**

**Table S1.** Performance comparison of depression presence classification models**.**

| Feature selection method | | Filter |  | Wrapper |  | Without feature selection |  |
| --- | --- | --- | --- | --- | --- | --- | --- |
|  | | Accuracy | F1-score | Accuracy | F1-score | Accuracy | F1-score |
| **Classifier** | |  |  |  |  |  |  |
|  | XGBoost | **0.66** | **0.66** | 0.62 | 0.60 | 0.61 | 0.60 |
|  | KNN | **0.57** | **0.57** | 0.49 | 0.49 | 0.50 | 0.48 |
|  | SVC | **0.62** | **0.62** | 0.48 | 0.46 | 0.54 | 0.53 |

Table S1 shows the model accuracy and F1-scores for three classifiers, XGBoost, KNN, and SVC, across different feature selection methods: filter-based, wrapper-based, and without feature selection. The highest performance metrics for each classifier are highlighted in bold. Specifically, the XGBoost classifier with filter-based feature selection achieved the best overall accuracy at 66% and an F1-score of 0.66. The results show that feature selection with a feature filtering method benefits all classifier models.

**Table S2.** Performance metrics for depression presence classification: Non-Depressed vs. Depressed.

| Metric | | Precision | Recall | NPV | F1-score | Support, n |
| --- | --- | --- | --- | --- | --- | --- |
| **Depression Severity** | |  |  |  |  |  |
|  | Non-depressed | 0.63 | 0.73 | 0.71 | 0.67 | 99 |
|  | Depressed | 0.71 | 0.61 | 0.63 | 0.65 | 109 |
| **Average** | |  |  |  |  |  |
|  | Macro average | 0.67 | 0.67 | 0.67 | 0.66 | 208 |
|  | Weighted average | 0.67 | 0.66 | 0.67 | 0.66 | 208 |

Table S2 summarizes the performance metrics of the XGBoost model for depression presence classification. The model demonstrates a moderate accuracy of 66% across 208 test samples. The 'Support' column indicates the number of instances for each class in the test dataset. For 'Non-Depressed ', the precision is 0.63, indicating that 63% of predictions for 'Non-Depressed ' were correct, while the recall of 0.73 shows that 73% of actual 'Non-Depressed ' cases were correctly identified. The NPV value of 71% means that when the model predicts someone is not depressed, there is a 71% chance they are genuinely not depressed. The F1-score for 'Non-Depressed ' is 0.67, affected by the lower precision score. For 'Depressed ', the model achieves a precision of 0.71, a recall of 0.61, an F1-score of 0.65, and an NPV of 0.63. The table also shows macro and weighted averages for precision, recall, F1-score, and NPV, ranging between 0.66 and 0.67, indicating a balanced performance between the two classes. Overall, the model exhibits a marginally higher accuracy in correctly identifying 'Non-Depressed ' cases than 'Depressed ' cases. The precision and recall metrics imply that the model is more conservative in predicting instances of depression. However, the moderate NPV values for both classes indicate room for improvement, especially in minimizing false negatives, which would be critical in a clinical setting.

**Table S3.** Comparative performance of depression presence classification models with biweekly PHQ-9 score as a predictor.

| Feature selection method | | Filter |  | Wrapper |  | Without feature selection |  |
| --- | --- | --- | --- | --- | --- | --- | --- |
|  | | Accuracy | F1-score | Accuracy | F1-score | Accuracy | F1-score |
| **Classifier** | |  |  |  |  |  |  |
|  | XGBoost | **0.82** | **0.82** | 0.79 | 0.79 | 0.77 | 0.77 |
|  | KNN | 0.74 | 0.73 | **0.76** | **0.76** | 0.63 | 0.57 |
|  | SVC | 0.73 | 0.71 | **0.80** | **0.80** | 0.71 | 0.70 |

Table S3 shows the model’s accuracy and F1-scores for three classifiers, XGBoost, KNN, and SVC, across different feature selection methods: filter-based, wrapper-based, and without feature selection. The highest performance metrics for each classifier are highlighted in bold. Specifically, the XGBoost classifier with filter-based feature selection achieved the best overall accuracy at 82% and an F1-score of 0.82. The results show that all models benefit from feature selection.

**Figure S1.** ROC curve for depression presence classification, showing an area under the curve (AUC) of 0.91. A high AUC value indicates a strong ability of the model to discriminate between individuals with and without depression.


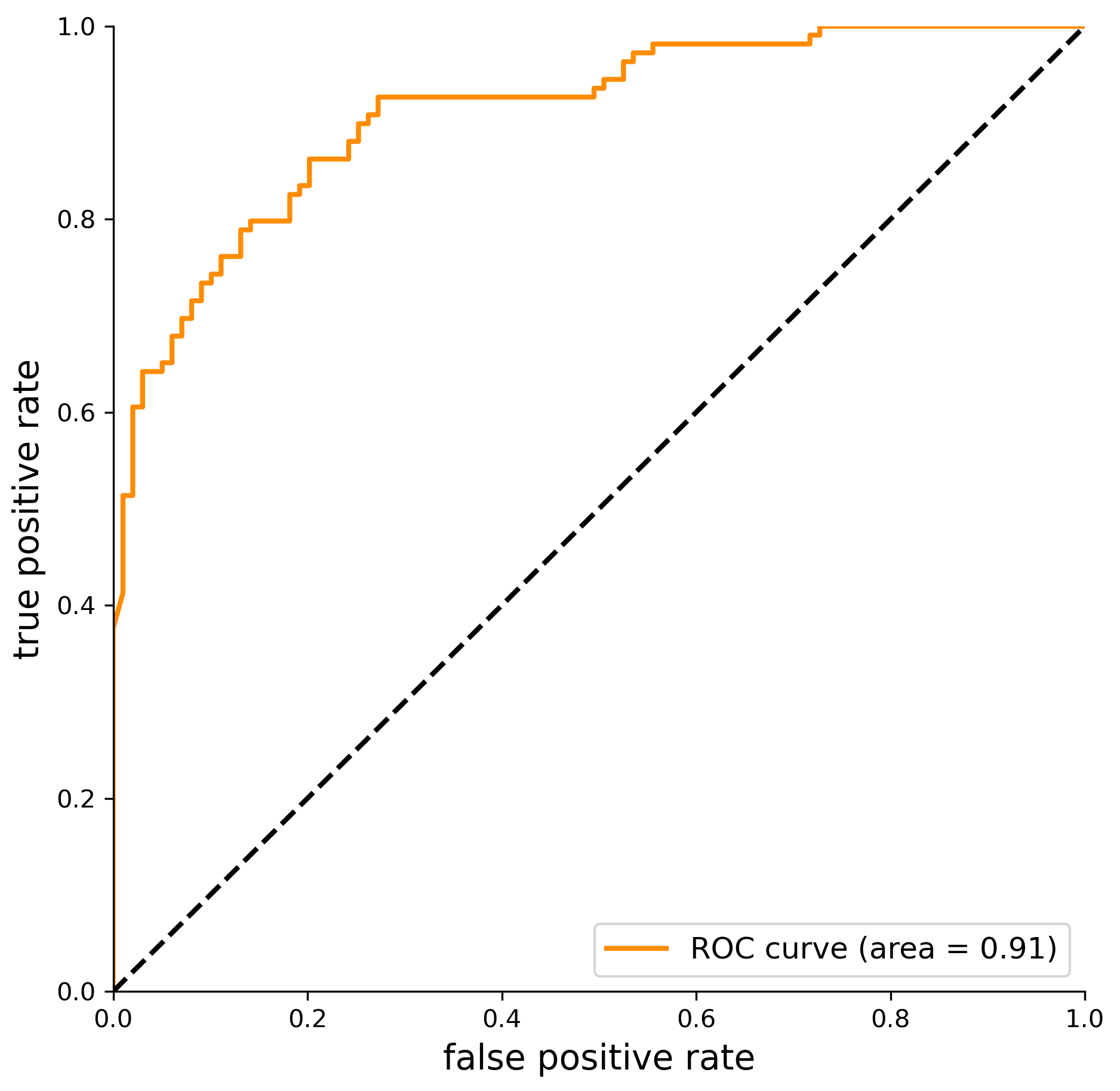


Figure S1 shows the ROC curve for depression presence classification using the XGBoost classifier. showing an area under the curve (AUC) of 0.91. A high AUC value indicates a strong ability of the model to discriminate between individuals with and without depression.

**Figure S2.** The confusion matrix for depression state transition classification.


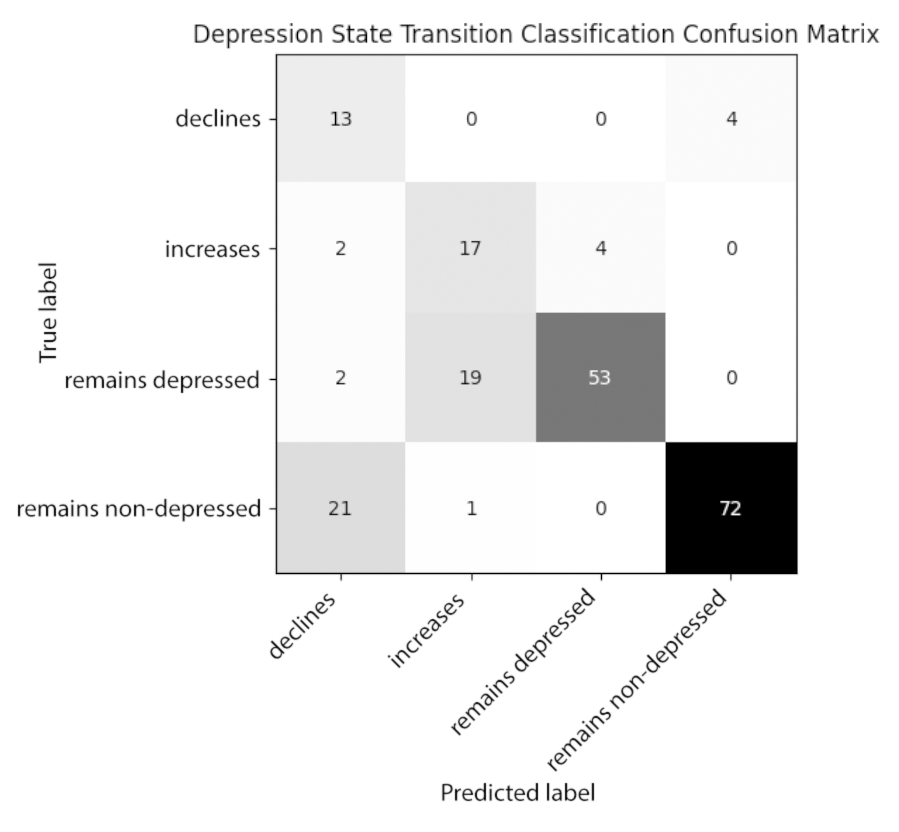


Figure S2 shows a confusion matrix representing the validation results of a model used for classifying depression state transitions. The matrix displays the validation results for the model classifying depression state transitions, contrasting true labels (shown on the y-axis) with the model's predictions (on the x-axis). The matrix cells display the count of predictions for each state, with diagonal cells representing correct predictions. While the matrix illustrates the model's ability to correctly identify most of the 'remains depressed' and 'remains non-depressed' cases, it also reveals a tendency to misclassify them as other transitions. Both 'declines' and 'increases' states show some misclassifications, reflecting challenges in the model's performance with less frequent states.

**Figure S3:** ROC curves for different depression transition classifications, showing areas under the curve (AUC).


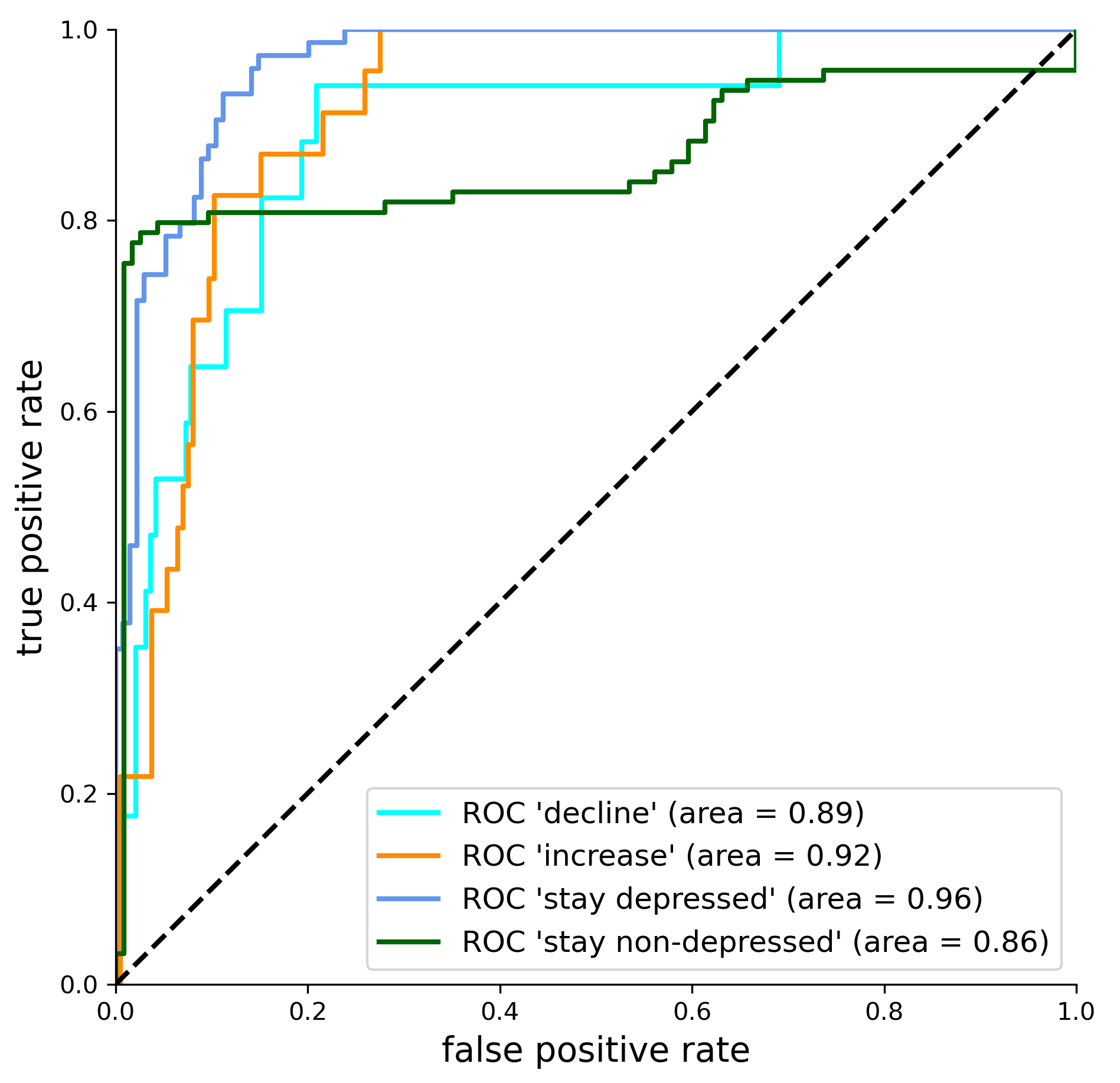


Figure S3 shows the ROC curves for depression transition cöassifications. The model performs best in identifying those who 'stay depressed' with the highest AUC of 0.96, followed by 'increase' (AUC = 0.92), 'decline' (AUC = 0.89), and 'stay non-depressed' (AUC = 0.86). These high AUC values indicate a strong ability of the model to discriminate between different depression transition states, with the highest accuracy in identifying sustained depression.
