# Supplementary material for: Predicting and Monitoring Symptoms in Patients Diagnosed With Depression Using Smartphone Data: Observational Study
Source: J Med Internet Res. 2024 Dec 3;26:e56874. doi: 10.2196/56874 (PMC11653032; doi:10.2196/56874)
Supplement: Multimedia Appendix 7 [file jmir_v26i1e56874_app7.docx]

## **Multimedia Appendix 7**

**Figure S1*.*** Key features in depression presence XGBoost classification model based on SHAP values**.**


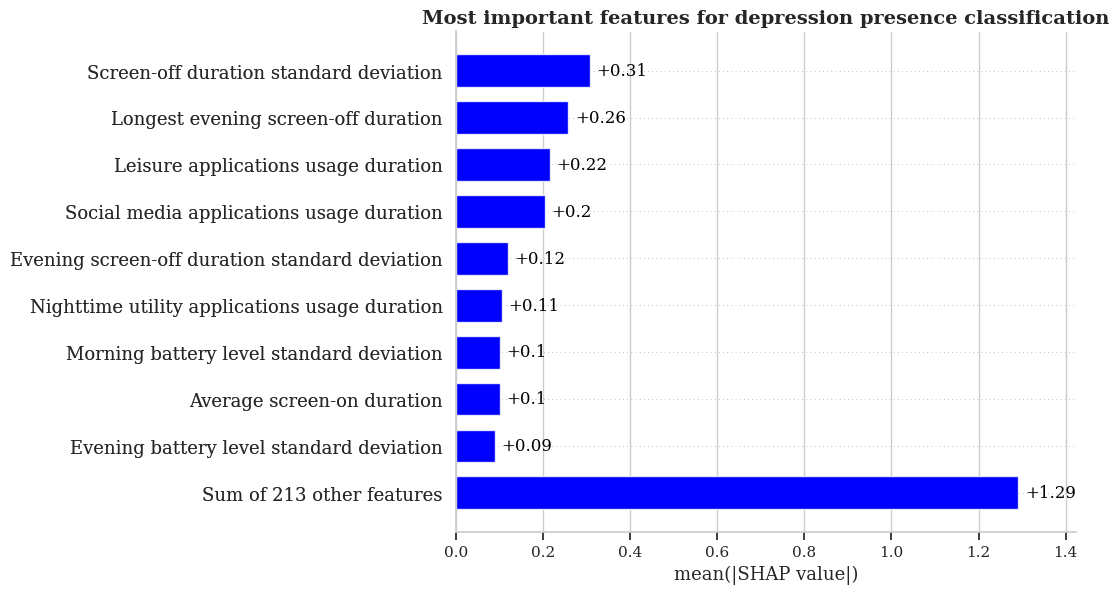


Figure S1 presents a bar chart ordering the features by the magnitude of their average impact on the model's predictions, with a higher mean SHAP value indicating a higher influence on the model's output. 'Screen-off duration standard deviation' (indicating the fluctuation in smartphone screen-off durations) is the most important feature, followed by 'Longest evening screen-off duration' (indicating the longest period of smartphone screen turned off during the evening), 'Leisure applications usage duration' (indicating the time difference between the notifications created by smartphone applications categorized as Leisure), and 'Social media applications usage duration' (similarly, indicating the time difference between the notifications created by smartphone applications categorized as Social media.). The 'Sum of 213 other features' at the bottom aggregates the importance of all remaining features. The SHAP values here reflect each feature's average impact on predictions rather than indicating whether a feature's impact increases or decreases the likelihood of depression according to the model. It is important to note that these SHAP values indicate each feature's relative importance in this model's decision-making process and are not directly comparable to SHAP values from other models.

**Figure S2.** Key features in depression presence classification XGBoost model with preceding biweekly PHQ-9 score as a predictor.

### **
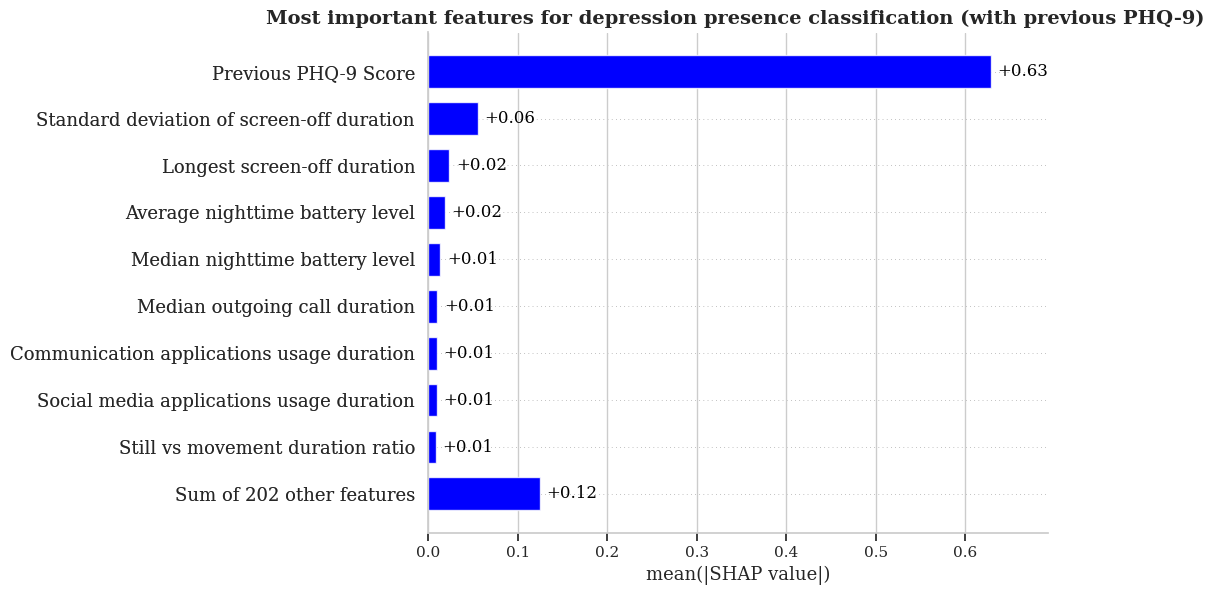
**

Figure S2 presents a bar chart illustrating the most important features of a depression presence classification model (previous biweekly PHQ-9 score added as predictor), as evaluated using SHAP values. The 'Previous PHQ-9 Score' is the most impactful feature, followed by 'Standard deviation of screen-off duration' (indicating the fluctuation in smartphone screen-off durations), 'Longest screen-off duration' (indicating the longest period of smartphone screen turned off during the evening), and 'Average nighttime battery level' (indicating the average smartphone battery level during the night). The chart's base shows the collective impact of 202 other features. These SHAP values denote the average contribution of each feature to the model's predictions without specifying their direction of impact on depression likelihood. It is important to note that these SHAP values indicate each feature's relative importance in this model's decision-making process and are not directly comparable to SHAP values from other models.

**Figure S3.** Key features in depression state transition classification XGBoost model with previous PHQ-9 score.


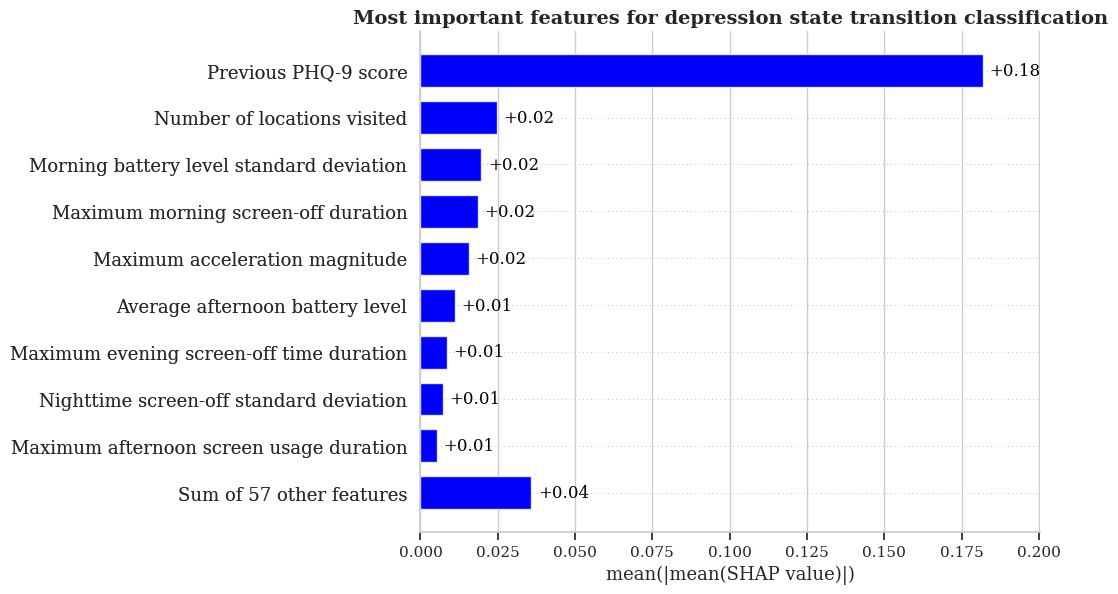


Figure S3 presents a bar chart illustrating the most important features for a depression state transition classification model (previous PHQ-9 score added as a predictor). The 'Previous PHQ-9 score' emerges as the most important feature, followed by 'Number of locations visited' (indicating the count of frequently visited places), 'Morning battery level standard deviation' (indicating fluctuations in the smartphone battery level during the morning), 'Maximum morning screen-off duration' (indicating the longest duration the mobile phone screen was off during the morning), and 'Maximum acceleration magnitude' (indicating the highest acceleration measured by the smartphone accelerometer). The features not explicitly shown in the table, indicated by the 'sum of 57 features', collectively have a less pronounced yet aggregated impact on the model's predictions. These SHAP values denote the average contribution of each feature to the model's predictions without specifying their direction of impact on depression likelihood. It is important to note that these SHAP values indicate each feature's relative importance in this model's decision-making process and are not directly comparable to SHAP values from other models.
